# Cartilage-hair hypoplasia: A comprehensive review

**DOI:** 10.70962/jhi.20250142

**Published:** 2025-10-01

**Authors:** Svetlana Vakkilainen

**Affiliations:** 1 Children’s Hospital and Pediatric Research Center, University of Helsinki and Helsinki University Hospital, Helsinki, Finland

## Abstract

Cartilage-hair hypoplasia (CHH) is a rare syndromic inborn error of immunity, caused by variants in the noncoding RNA gene *RMRP*. The effects of *RMRP* deficiency are pleiotropic, affecting the ribosomal RNA processing, cell cycle, and gene regulation. Typical clinical manifestations of CHH include chondrodysplasia with short stature, hair hypoplasia, combined immunodeficiency, and anemia. In addition, individuals with CHH have increased prevalence of malignancies, Hirschsprung’s disease, and autoimmunity. The only curative option for immunodeficiency or severe anemia in CHH remains hematopoietic stem cell transplantation. This review summarizes 60 years of CHH research, covering genetic aspects, pathogenesis, clinical and laboratory features, as well as diagnostic and management considerations in CHH.

## Teaser

This comprehensive review of cartilage-hair hypoplasia (CHH) by Vakkilainen summarizes 60 years of CHH research, covering genetic aspects, pathogenesis, clinical and laboratory features, as well as diagnostic and management considerations in this rare syndromic inborn error of immunity.

## Graphical Abstract

**Figure:**
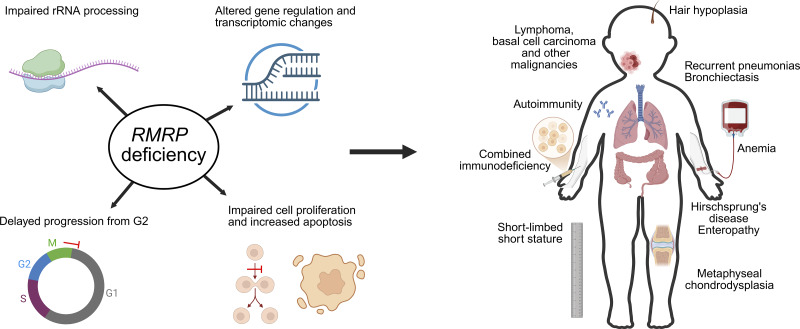

## Introduction

Cartilage-hair hypoplasia (CHH), a specific form of metaphyseal dysplasia, was initially reported by Maroteaux et al. in 1963 (1). Two years later, a series of 77 Amish patients was published by McKusick et al. describing the metaphyseal cartilage hypoplasia in the long bones and expanding the phenotype to include hair hypoplasia, malabsorption, congenital megacolon, and fatal varicella (2). In 1970s, the laboratory features of immunodeficiency, including decreased counts and function of lymphocytes, were recognized (3, 4, 5). CHH was found to be enriched in the Finnish population (6), with an estimated incidence of 1 in 1,340 in Amish and 1 in 23,000 births in Finnish (7, 8). Finally, *RMRP* gene was discovered as the cause of CHH in 2001 by Ridanpää et al., who mapped it to the chromosome 9p13 (9).

Currently, *RMRP* deficiency is perceived as a clinical spectrum, ranging from severe anauxetic dysplasia (AD) or severe combined immunodeficiency (SCID) to mildly affected patients. Some cases have been deemed to have “metaphyseal dysplasia without hypotrichosis,” formerly a distinct disease entity (10). However, the longitudinal follow-up of such individuals has revealed late-onset development of immune abnormalities typical for CHH (11).

In the recent decades, much has been learned about *RMRP* deficiency. This review summarizes the genetics, molecular pathogenesis, clinical features, and management of CHH.

## Genetic background

**Figure 1.**
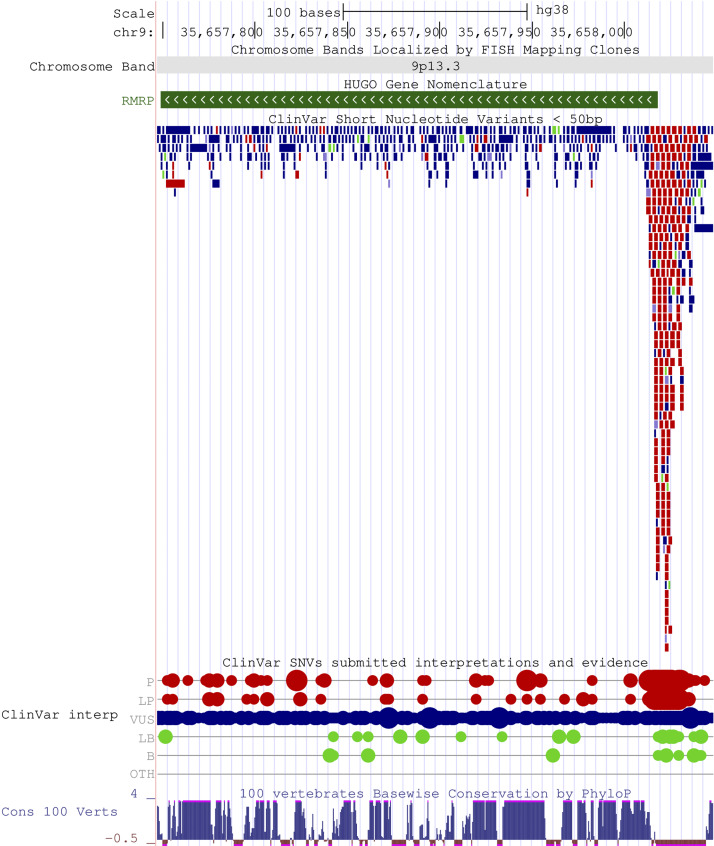
**Visualization of variants with ClinVar interpretation, as well as evolutionary conserved regions of *RMRP*.** The figure was created using the UCSC Genome Browser (12); the stable link is https://genome-euro.ucsc.edu/s/Integriin/hg38_RMRP.

*RMRP* is a 269-nucleotide long gene encoding the long noncoding RNA (Fig. 1). Following updates in the National Center for Biotechnology Information human genome reference sequence, the nomenclature of nucleotides has shifted. For example, the founder variant in the Finnish and Amish populations (n.71A>G) has been previously reported as n.70A>G and most recently as n.72A>G. Throughout this review, the reference sequence NR_003051.3 is used.

The founder variant n.71A>G arrived in Finland ∼4,000 years ago, before the hypothesized time of the population expansion, and CHH belongs to the Finnish disease heritage (13). The carrier rates of the founder variant are 1:19 and 1:76 among Amish and Finnish individuals, respectively (7). In the Japanese population, two founder variants have been identified: a 17-bp duplication in the promoter region and n.219A>G (14, 15). In Brazilian population, n.196C>T has been described as the founder variant (16).

*RMRP* is highly conserved across species (17, 18). Pathogenic variants in the transcribed region are located throughout the gene, but mostly in the highly conserved nucleotides, whereas benign variants preferentially locate at evolutionary non-conserved nucleotides (Fig. 1) (18). Variants in the promoter region imply insertions, duplications, or triplications localized between the TATA box and the transcription initiation site. A combination of two pathogenic variants in the promoter region of *RMRP* has only rarely been observed in humans (19, 20, 21). All three reported cases exhibited a severe immunodeficiency phenotype. This, together with failure to produce *RMRP* knockout yeast or mice, suggests that *RMRP* expression might exhibit a dose-dependent phenotype correlation and that complete *RMRP* deficiency is lethal.

Importantly, pathogenic variants in *RMRP* may be missed on whole-exome sequencing; therefore, *RMRP* coverage should be specifically inquired for (11, 22). Alternatively, diagnostic approach can include whole-genome sequencing or targeted panels that fully capture the *RMRP* gene, including the promoter region, as well as copy number variation assessment.

Another diagnostic challenge relates to *RMRP* variant interpretation because the American College of Medical Genetics and Genomics criteria for variant classification should be adapted to this noncoding gene. The absence of translated protein, the paucity of functional in vitro assays, and phenotypical variability prevent implicating many of the classifying criteria. As an example, of the four criteria of strong evidence of pathogenicity (PS1-4), none is applicable to *RMRP* variants (23). Fortunately, the ClinGen Curation working group has been recently established for the ongoing *RMRP* variants’ interpretation.

## Molecular and cellular functions of *RMRP*

### Mitochondrial RNA processing complex

**Figure 2.**
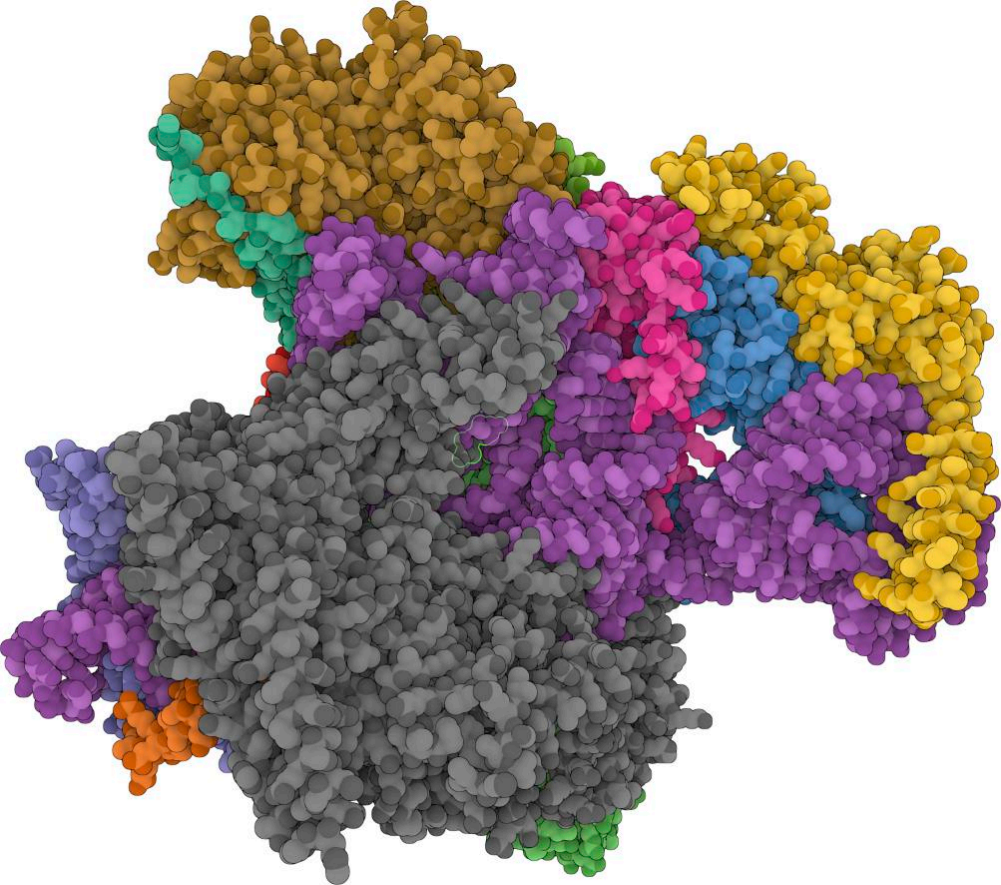
**The predicted structure of human mitochondrial endoribonuclease complex, consisting of 11 protein subunits and one RNA subunit (*RMRP*, in violet).** The position of the Finnish and Amish founder pathogenic variant n.71A>G is marked with light green borders, localizing in the very heart of the complex. The visualization was created using the AlphaFold Server (36) and is subject to AlphaFold Server Output Terms of Use (https://alphafoldserver.com/output-terms).

*RMRP* is an RNA component of the ribonuclease for mitochondrial RNA processing (MPR). MPR was first identified as a target of autoantibodies from sera of patients with systemic lupus erythematosus and related rheumatic diseases (24, 25). This endoribonuclease is abundant in eukaryotic cells and contains several protein subunits (including Rpp14, Rpp20, Rpp25, Rpp29, Rpp30, Rpp38, Rpp40, Pop1, and Pop5) (26, 27). All of these proteins are shared with RNase P, which is involved in pre-transfer RNA (tRNA) processing in eukaryotes and prokaryotes (26), complicating studies on MPR, which precipitates mostly together with RNase P (28). Only very recently *RMRP*-specific human protein subunits (Rmp24 and Rmp 64) have been identified by a genome-wide screening (27). The precise structure of human MPR is yet to be finally described (29, 30), but the AlphaFold Server prediction is shown in Fig. 2. Several studies have investigated the effects of mutated *RMRP* on MPR stability, demonstrating impaired binding of *RMRP* to various protein subunits (28, 31, 32). Substrates for MRP include (1) mitochondrial replication RNA primer, which is also cleaved by RNase P (25), (2) pre-ribosomal RNA (rRNA) (33), (3) cell cycle cyclin *clb2* mRNA (34), as well as (4) viperin (*RSAD2*) mRNA (35).

*RMRP* is widely expressed in all human tissues tested (37). As *RMRP* is an untranslated RNA, functional testing of variants has been challenging and is not routinely available. However, reduced *RMRP* expression has been demonstrated in numerous *RMRP*-deficient cell lines, yeast and zebrafish models, as well as in patient-derived cells (Table S1) (21, 28, 31, 37, 38, 39, 40, 41, 42). While the expression of *RMRP* can be measured by RT-PCR for diagnostic purposes (21), severe lymphopenia may skew the results. Also, it remains unknown to what extent, if at all, other defects in cell function, including other etiologies of SCID, affect *RMRP* expression levels. Therefore, decrease in *RMRP* expression in immunodeficient patients has yet to be shown to be specific to CHH. One approach for functional testing of *RMRP* deficiency may be to measure the accumulation of MPR substrates. In ATDC5 cell line, silencing of *Rmrp* by siRNA resulted in significantly reduced levels of *Rmrp* expression, as well as elevated levels of *Clb2* mRNA, *Viperin* mRNA, and internal transcribed spacer (ITS)1 pre-rRNA (40). The increase of ITS1 pre-rRNA processing intermediate has been demonstrated also in dermal fibroblasts from patients with CHH (40).

Of note, variants in the gene encoding one of the protein subunits of MRP, *POP1*, have been identified as a cause of a severe skeletal dysplasia in several patients (43, 44). The clinical and radiographic features of the affected siblings resembled AD with no extra-skeletal manifestations. *RMRP* expression was reduced in patients’ peripheral blood mononuclear cells (PBMC), consistent with previous studies demonstrating the reduction of *nme1* levels in yeast with *pop1* mutations, and implying the destabilization of MPR as underlying mechanism (43, 45). In addition, patients’ PBMC demonstrated impaired proliferation (43). In terms of the effects of *POP1* mutations on the rRNA cleavage, contradictory results have been reported (43, 45). These findings, together with other known ribosomopathies affecting the skeleton (*RPL13*, *SBDS*, and *NEPRO* gene defects), emphasize the pivotal role of ribosomes in skeletal development (46, 47, 48).

### Ribosomal RNA processing

Cellular functions of *RMRP* have first been described in yeast, a model organism for rRNA processing. Mouse model for *RMRP* deficiency has never been established, as *Rmrp* knockout by insertion of DNA elements upstream the promoter was lethal early in embryonic development before embryonic day (E) 6.5 (49). The deletion of *nme1* gene, the *RMRP* ortholog in yeast, is also lethal (17); therefore, various mutant strains have been used for further experiments, demonstrating the impaired procession of 5.8S rRNA (Table S1) (33, 37, 50). Later, similar evidence of abnormal cleavage of pre-rRNA in the ITS1 region has been found in cell lines and patient-derived fibroblasts (31, 51).

### Mitochondrial function

*RMRP* was initially isolated from mitochondria (52) but had later been shown to localize mostly in the nucleolus (53). In yeast *nme1* mutants, mitochondrial function was described as unaffected; however, the method of measurement has not been reported (37). Apart from cleavage of the mitochondrial replication primer, *RMRP* binds to two RNA-binding proteins, HuR and GRSF1, that facilitate *RMRP* nuclear export and contribute to its accumulation in the mitochondria, respectively (54). In HeLa cells with a 45% decrease in *RMRP* expression, Seahorse analysis revealed decreased basal oxygen consumption rate (54). The role of *RMRP* in mitochondrial function thus remains poorly characterized and deserves further studies.

### Cell cycle and apoptosis

Defective cell proliferation has been an early recognized feature in T and B lymphocytes and fibroblasts from patients with CHH (55) and has since been demonstrated in yeast, zebrafish, various cell lines, and repeatedly in patient cells (Table S1) (31, 34, 39, 40, 42, 50, 51, 55, 56, 57, 58, 59). *RMRP*-deficient cells accumulate in the G2 phase of cell cycle and show altered expression of several major cell cycle cyclins and kinases. In addition, patients’ PBMC exhibit increased apoptosis (58, 60). Defective lymphocyte proliferation is the most clear disease mechanism in CHH, connecting multiple molecular abnormalities with clinical features, including increased susceptibility to infections, autoimmunity, and malignancies.

Transcriptomic differences in primary dermal fibroblasts from patients with CHH compared with healthy controls have been reported in two independent studies (41, 59). These included dysregulated expression of genes related to cell cycle pathways, cellular movement, cellular development, cell death and survival, as well as cellular growth and proliferation. In addition, transcriptional profile from peripheral blood leukocytes of two CHH patients showed 99 upregulated and 38 downregulated genes, participating in cell cycle regulation and apoptosis (including upregulated *G0S2*), as well as signal transduction (37).

### Telomere biology

In HeLa and 293T cell lines, *RMRP* has been shown to interact with telomerase reverse transcriptase (TERT) (61). While TERT–*RMRP* complex did not exhibit telomerase activity, it did show RNA-dependent RNA polymerase activity, which is known to be involved in the posttranscriptional gene silencing. Moreover, TERT–*RMRP* complex produced double-stranded *RMRP* in vitro and in vivo, and *RMRP* expression was controlled by negative feedback via *RMRP*-specific siRNA.

These findings led to telomere biology studies in primary CHH cells (42, 62). Telomere length has been assessed by flow-FISH in white blood cell subsets from 15 patients with homozygous n.71A>G *RMRP* variants (42). In 11 of them, telomeres were shorter in lymphocytes compared with normal telomere length in healthy heterozygous n.71A>G variant carriers. Telomere length was also low in granulocytes in seven patients. Additionally, telomerase activity in patients’ stimulated lymphocytes was significantly lower compared with healthy controls, while *TERT* and *TERC* expression in the peripheral blood lymphocytes was not different. In a cohort of 48 Finnish patients with CHH, their healthy relatives carrying *RMRP* variants, and healthy controls, relative telomere length was measured by quantitative PCR from peripheral blood DNA samples (62). Altogether, 52% of all patients, and specifically 89% of children with CHH, demonstrated short telomere length for age, translating into an absence of negative correlation between telomere length and age. No association between telomere length and clinical features was detected.

### Gene regulation

In a study implementing various cell lines, *RMRP* processing differed according to cell type and produced small RNAs that silenced large sets of genes (63). Moreover, the presence and amount of these silencing *RMRP*-derived RNAs varied during cell differentiation. The enriched genes regulated by these RNAs covered several pathways, including cell death, cell cycle, as well as connective tissue and hematologic system development, among others. These findings underscore the incomprehensibly pleiotropic effects of *RMRP* deficiency.

### Chondrodysplasia mechanisms

Histological examination of the patient’s bone back in 1960s demonstrated the paucity of cartilage cells and disturbed columnar tissue organization (2). The role of *RMRP* in cartilage development has since been investigated in detail, and the pathogenesis of chondrodysplasia in CHH has been studied in various in vitro models (Table S1).

*Rmrp* expression and the distribution of MPR protein subunits have been explored in the growth plate of 6-wk-old mice, demonstrating that they co-clustered mostly to the hypertrophic zone chondrocytes of the developing growth plate (40).

In *rmrp* knockout zebrafish model, chondrogenesis was disrupted and bone ossification was delayed (39). The developmental cartilage abnormalities showed disordered chondrocyte arrangement and resulted in abnormal pharyngeal arch pattering and shaping, recapitulating the CHH phenotype. This involved the upregulation of Wnt/β–catenin pathway, confirmed at the level of both RNA and protein expression. In addition, inhibition of Wnt/β–catenin signaling by a β-catenin inhibitor XAV939 partially alleviated the chondrogenic dysplasia in mutant zebrafish.

Dermal fibroblasts from patients with CHH and healthy control were trans-differentiated toward a chondrocyte-like phenotype (40, 41). The engagement of CHH fibroblasts into the transdifferentiation process was delayed, and terminal differentiation was disrupted. The expression, as well as protein level of certain growth factors involved in the regulation of chondrogenic differentiation, was altered in CHH chondrocytes.

## Clinical features of *RMRP* deficiency and management considerations

Hemizygous mice missing one *RMRP* allele showed 50% decrease of *RMRP* expression in embryonic fibroblasts and were healthy (49). In accordance, humans carrying pathogenic variants in a single allele remain asymptomatic (8, 64) and do not demonstrate cell proliferation defects (42).

Genotype–phenotype correlation has not been consistently demonstrated in CHH. Siblings with identical pathogenic variants can exhibit dramatically different clinical course of immunodeficiency (19, 65). All three case reports of homozygous variants in the promoter region of *RMRP* associated with severe phenotype (19, 20, 21). A study of human fibroblasts transfected with various *RMRP* pathogenic variants analyzed rRNA and certain mRNA expression levels and searched for genotype–phenotype associations (66). Negative correlation was observed between the severity of bone dysplasia and rRNA cleavage activity, as well as between the severity of immunodeficiency/anemia or the presence of hair hypoplasia and *CCNB2* mRNA cleavage activity. Finally, of the 36 subjects with CHH who underwent hematopoietic stem cell transplantation (HSCT) and whose genotype has been reported, only nine were homozygous for the n.71A>G variant, suggesting that this genotype might be less common in severely affected patients (19, 67, 68, 69).

### Chondrodysplasia

Chondrodysplasia in CHH leads to disproportional short stature, with short extremities, affecting mostly metaphyses but also epiphyses. Typical features in addition to short limbs include short hands, incomplete extension of elbows (present in 92%, 79/86 patients) despite overall ligamentous laxity (95%, 81/85), lower limbs varus deformity (63%, 54/86), chest deformity (68%, 57/84), as well as lumbar lordosis (85%, 72/85) and scoliosis (21%, 18/86) (65, 70). The review of orthopedic data from 135 North American patients with CHH revealed that ∼43% had required surgical realignment for the bowing of lower extremities (70).

A review of radiologic changes in 82 Finnish patients with CHH demonstrated that the growth failure of long bones was obvious from infancy and was progressive, particularly in femur (71). In most of the children studied, the costochondral junctions were wide and prominent. Radiologically, metaphyseal abnormalities included flaring, cupping, widening, cysts, fragmentation, and scalloping of metaphyses in the tubular bones, particularly at the knee. Epiphyseal changes could be seen in hand and foot bones.

Importantly, metaphyseal changes may not be apparent in the first 2 years of life (72, 73). Some patients’ radiographs show no signs of metaphyseal dysplasia despite evident short stature (19) or severe immunodeficiency (67). In addition, after the epiphysis closes, the metaphysis may remain defective or may be normal (65, 74).

### Short stature

CHH-specific growth charts assist in longitudinal follow-up (75). Short stature in CHH is mostly prenatal, with birth length corrected for gestational age being −2.9 standard deviation score (SDS) for boys and −3.0 SDS for girls (75). In the Finnish and in the ethnically heterogenous cohort, 70% and 64% of patients, respectively, were born short, with birth length below −2.0 SDS (65, 76). However, in the Japanese CHH cohort, prenatal growth failure was infrequent, as none of the nine patients with data available regarding gestational age was born short (15). The growth failure is progressive, particularly during the first year of life and during puberty, resulting in the median adult height of 131 cm for males and 123 cm for females (75).

While short stature was first thought to be invariably present in CHH patients, individuals with normal height have since been reported (15, 19, 77). In particular, compound heterozygosity with promoter region duplications or triplications has been associated with milder growth retardation in CHH (19, 77). Normal growth during childhood can still associate with severe extra-skeletal features, including lethal lymphoma in a 6-year-old child (19, 77).

Growth hormone (GH) treatment in CHH is mostly considered futile. Several case reports (altogether, of eight patients) have described GH therapy as ineffective or, at most, with temporary benefit (11, 15, 22, 78, 79). However, GH deficiency has also been reported in CHH patients, and for them, GH treatment might be beneficial (15, 18, 80). In addition, in a single patient with genetically confirmed CHH and no GH deficiency, GH treatment improved height from −4.2 SDS prior to treatment at 3 years of age to −2.6 SDS at 6 years (81).

### Anemia

In a cohort of 88 Finnish patients, low hemoglobin levels were common in childhood (54/74, 73%), in addition to macrocytosis (in 47%), but were not seen in adult subjects (82). In addition, macrocytosis has been observed in 50% (10/20) patients with no anemia. Reticulocyte index and haptoglobin, iron and transferrin concentrations, as well as vitamin B12 and folate levels were normal. Three deaths from pneumonia and/or sepsis were described in this historical cohort, all in infants with profound anemia. Anemia is associated with neutropenia, more severe growth failure, and weaker lymphocyte proliferative responses to phytohemagglutinin.

Another study described 12 patients with CHH and severe anemia, defined as hemoglobin levels below 30 g/liter or a history of repeated red blood cell transfusions (83). All but one child developed anemia in the first 4 mo of age, while a single patient became anemic at 2.5 years of age. The same distribution of age at onset of anemia has been demonstrated in a more recent study (84). Of note, all children had very severe short stature (height from −5.5 to −9.9 SDS).

Bone marrow erythroid hypoplasia and poor growth of erythroid precursors have been demonstrated in all CHH patients with severe anemia who underwent bone marrow examinations (22, 83, 85). Importantly, erythroid hypoplasia may not be obvious early in the course of severe anemia in CHH but ultimately becomes evident later (83). Bone marrow cultures showed severely impaired erythroid colony formation even in those with normal hemoglobin concentrations (86, 87). Granulocyte-macrophage and megakaryocyte growth was also remarkably defective, despite normal neutrophil and platelets counts in the peripheral blood.

The incidence of transfusion-dependent anemia in CHH has increased in Finnish patients in the 2000s, from 6% (7/114) in older reports to 31% (10/32) in the most recent publication (84). This may reflect a better recognition of hematological manifestations of CHH translating into screening for anemia, as well as improved survival early in life. Contrary to earlier reports, in Finnish patients born in the 2000s, severe anemia did not associate with the severity of short stature or the degree of T cell deficiency.

Apart from hypoplastic anemia, autoimmune hemolytic anemia (AIHA) has also been described in multiple patients and is the most common autoimmune phenomenon in CHH (18, 68, 84, 88, 89, 90, 91).

The natural history of severe hypoplastic anemia in CHH may show spontaneous resolution in infancy but also prolonged (up to 5 years) periods of remission, with then reoccurring transfusion dependency (82, 83, 85). The only curative option for severe anemia in CHH remains HSCT, with multiple case reports of successful resolution (22, 83, 84).

Unsuccessful therapies for CHH hypoplastic anemia include GH (*N* = 3) (85), androgen (82), and erythropoietin (*N* = 1) (22), whereas steroid therapy has demonstrated variable results (83). Treatment with sirolimus resolved anemia and reversed bone marrow erythroid hypoplasia and the growth defect of erythroid precursors in a single subject with CHH (22). The interruption of sirolimus led to relapse of anemia, and the reinitiation led to resolution once again. This report raises the question of the effect of dysregulated immune environment in the CHH bone marrow, which may result in anemia and may be reversible with immunomodulation.

### Hirschsprung’s disease and enteropathy

Gastrointestinal complaints, most frequently prolonged and/or recurrent diarrhea, are common in subjects with CHH, reported in 32/104, 31% of the Finnish cohort (92). Gastroscopy can demonstrate chronic gastritis, peptic ulcers, and/or duodenal villous atrophy. Case reports have described nonspecific inflammatory enteropathy in patients with chronic diarrhea and malabsorption requiring parental nutrition (65, 79). Consistent with these findings, the *rmrp* knockout zebrafish model exhibited a hypoplastic gastrointestinal tract and smaller intestine with fewer folds, intestinal epithelial cell numbers were decreased, and intestinal villi were absent (30).

Hirschsprung’s disease (HD) is a well-known comorbidity of CHH, although the pathogenetic link between *RMRP* deficiency and HD remains unknown. The prevalence of HD in the Finnish CHH cohort has increased from 9% (13/142) in the 20^th^ century to 25% (8/32) in the 2000s (84, 93). This increase can be explained by previously undiagnosed early fatal cases. Historically, CHH-HD has been associated with more severe growth failure, complete alopecia, and severe anemia (93). Out of 13 historical HD cases, five patients had deceased, four of them due to severe infection, described as enterocolitis-related septicemia (93). However, in the 2000s cohort, HD did not correlate with shorter birth length or severe anemia (84). Moreover, children with HD had less infectious complications (84). Whether these findings represent an improved surgical, nutritional, and anti-infection management in HD remains to be demonstrated.

### Malignancies

Increased cancer risk was reported in 1999 in a cohort of 122 Finnish patients with CHH compared with the general Finnish population, while this was not observed in their unaffected siblings or parents (64). The standardized incidence ratio (SIR) for all malignancies was 6.9, mostly arising from a SIR of 90 for non-Hodgkin’s lymphoma. These findings were updated in 2008, also identifying SIR of 33 for basal cell carcinoma (94). The most affected age group was young adults, with SIR of 130 for non-Hodgkin’s lymphoma in patients aged 15–29 years. Malignancies in this cohort were associated with poor prognosis, resulting in death of nine out of 14 patients with non-skin cancers. Other types of malignancies in CHH include carcinomas of thyroid, larynx, vocal cord, bile duct, or lip, testicular progonoma, leukemia, Hodgkin’s and other types of lymphomas, plasmacytoma, or myelodysplasia (64, 95).

In a case series of lymphomas in 16 Finnish CHH patients, most were diffuse large B cell lymphomas (DLBCL) (6/16, 38%) and diagnosed in advanced stage, resulting in 11 deaths (69%, 11/16) (96). Molecular testing for Epstein-Barr virus (EBV) was performed in three patients and was positive in all of them, in two from malignancy samples and in one from blood.

Importantly, malignancy can develop in patients with clinically inapparent immunodeficiency, as demonstrated in a prospective follow-up study in which eight of the 15 CHH individuals with non-skin cancer had no preceding clinical symptoms of immunodeficiency (95). Malignancies are a significant contributor to CHH-related mortality, with the observed standardized mortality ratio (SMR) of 8.8 for all malignancies, primarily lymphoid/hematopoietic malignancies with SMR of 60 (95).

Regarding the prevention and early diagnosis of malignancy in CHH, low threshold for imaging and more invasive diagnostic workup should become standard. All surviving lymphoma patients had been diagnosed with lymphoma either through routine ultrasound screening or after evaluation for nonspecific mild symptoms (95, 96). Skin cancer in CHH develops only on sun-exposed areas (95); therefore, enhanced sun protection should be emphasized.

### Infections

The definitions of increased susceptibility to infections vary among CHH studies, and the prevalence of severe infections is obviously higher in the reports describing transplant outcomes in CHH. In the clinical cohorts, 33–78% of the patients have normal infection history (Table S2). Apart from recurrent respiratory tract infections, severe and/or opportunistic infections described in CHH include severe varicella, pneumonia (*Pneumocystis jirovecii*, *Aspergillus* spp., and cytomegalovirus), hepatitis (adenovirus and human bocavirus), disseminated viral infections (adenovirus, cytomegalovirus, herpes simplex virus, human herpesvirus-6, EBV, and parvovirus), *Haemophilus influenzae* meningitis despite proper vaccination, chronic norovirus gastroenteritis, lethal enteroviral meningoencephalitis, refractory warts, as well as vaccine-strain rubella virus-induced skin granulomas (19, 20, 21, 65, 67, 68, 84, 88).

The first report on patients with CHH from the Amish community described fatal primary varicella (2). This has not been reported in later case series; vice versa, the majority of patients with CHH clear varicella without antiviral medications or any complications (65, 88, 97). However, at least seven hospitalizations due to varicella, including two confirmed cases of varicella pneumonia, have been described in a Finnish CHH cohort (97). Also, some patients have received post-exposure immunoglobulin prophylaxis or early antiviral therapy for varicella due to its notoriety as potentially fatal in CHH.

### Pulmonary manifestations

Severe progressive lung disease, with bronchiectasis and recurrent pulmonary infections, is an important cause of death in adults with CHH (95, 98, 99). Hypogammaglobulinemia is present in some, but not all, of these patients, and immunoglobulin replacement therapy (IGRT) is often insufficient to prevent deterioration. Bronchiectasis can develop early in children with CHH, as reported in several patients aged 6, 10, and 11 years (89, 90).

A retrospective study described 15 Finnish patients with CHH, aged 2–39 years, all with respiratory symptoms, who underwent chest high-resolution computed tomography (HRCT) imaging (*N* = 13) or bronchography (*N* = 2) (99). Bronchiectasis were detected in eight patients (8/15, 53%). All these patients had mostly severe respiratory symptoms and recurrent pneumonias and tended to have more severe growth failure compared with the general cohort of CHH patients.

In an unselected cohort of 34 Finnish patients, aged 13–68 years, 10 (29%) had bronchiectasis on chest HRCT imaging (100). Some also had fibrosis-like changes in the lungs. None had hypogammaglobulinemia, but several had specific antibody deficiency. Importantly, lung magnetic resonance imaging had a good correlation with HRCT and could be used for monitoring patients with CHH, sparing them from repeated radiation. In a follow-up study of these 34 patients, 14 were available and underwent lung magnetic resonance imaging at a median of 6.8 years since initial imaging (101). Bronchiectasis radiologic scores either improved or remained unchanged in all patients, despite some of them experiencing sinopulmonary infections during follow-up.

### Autoimmune diseases

Autoimmune diseases have been diagnosed in 11/104 (11%) of Finnish individuals with CHH, compared with the prevalence of 5% in the general Finnish population (92). The spectrum of autoimmunity in CHH is broad and includes more common conditions (AIHA, thrombocytopenia, and juvenile rheumatoid arthritis) and single cases of neutropenia, celiac disease, thyroid diseases, multifocal motor axonal neuropathy, narcolepsy, psoriasis, and ulcerative colitis.

As in many other inborn errors of immunity, autoantibodies are commonly found in CHH. Serum samples from 16 CHH patients were screened with IgG autoantibody microarray (102). Broad heterogenous reactivity was observed, similar to patients with autoimmune polyendocrinopathy-candidiasis-ectodermal dystrophy. The most significant autoantibodies were gliadin, Mi-2, myosin, PCNA, PL-7, Sm/RMP, SRP54, thyroglobulin, and vitronectin. No clinical symptoms or signs suggestive of specific autoantibody pathogenicity were reported. In another study, serum samples from 33 adults with CHH were assessed for the presence of seven autoantibodies related to celiac disease or common in autoimmune polyendocrinopathy-candidiasis-ectodermal dystrophy (92). Autoantibody reactivity was detected in 6/33 (18%) of the patients, with no compatible symptoms.

### Granulomatous inflammation

Several cases of inflammatory granulomas have been reported in CHH, mostly in severely immunodeficient patients. Granulomas may develop prenatally, as described in case of a fetus with extensive granulomatous inflammation in most of the tissues studied, including spleen, liver, lungs, and myocardium (103).

In a fully vaccinated CHH patient, progressive skin granulomas starting at ∼1 year and 8 mo of age have been reported (104). The skin biopsy showed monoclonal T cell infiltrate and some EBV-positive cells. The patient was severely immunodeficient with absent naïve T cells, had EBV viremia and developed an EBV-DLBCL abdominal mass. The treatment with rituximab and HSCT resulted in cure from lymphoma, T cell immunoreconstitution, and significant reduction in skin granulomas. In addition, a girl with CHH and facial cutaneous granulomas has been reported, who developed lung DLBCL (105). Despite chemotherapy for lymphoma, the skin lesions progressed. Lesions’ EBV testing was positive on repeated biopsies. Again, HSCT was performed and resulted in regression of cutaneous lesions.

In a case series, granulomatous inflammation was described in four patients with CHH, all with severe lymphopenia (89). The onset of skin granulomas was from the age of 12 mo to 8 years In one patient, granulomatous-destructive lesions manifested also in bones, lymph nodes, spleen, and nasal cartilage. Two children received HSCT, and their granulomas resolved 3–14 mo after HSCT. In another patient, spontaneous regression of granulomas was observed after 17 years.

The presence of rubella virus in granulomas has not been examined in the abovementioned cases. A case of vaccine-derived rubella virus has been reported in one non-Finnish patient with CHH, who was vaccinated at 18 years of age (106). Skin granulomas developed at 27 years of age, and other clinical features included DLBCL, AIHA, hepatosplenomegaly, as well as naïve T cell lymphopenia. Recently, vaccine-strain rubella virus induced skin granulomas in the most severely immunodeficient child from the Finnish cohort have been reported (84).

### Puberty and fertility

Pubertal development may be impaired in CHH, as demonstrated by a report of two cases of hypogonadism, one of a hypogonadotropic and another of normogonadotropic (107). These were managed with hormone therapy and underscore the significance of using CHH-specific growth charts for patient follow-up, as well as of multidisciplinary care in CHH.

Important health consideration in females with CHH is regular gynecological assessment. In a series of 18 unselected patients, in eight (44%, 8/18), human papillomaviruses were detected from cervical samples, and three of them also exhibited cell atypia in Pap smear (108). Fertility has not been reported to be impaired in females with CHH; however, single cases with low serum anti-Müllerian hormone levels have been described (107).

In a case series of 14 females with CHH, 42 pregnancies were reported (109). Most (25/42, 60%) were carried to term, including one twin delivery, and there were no complications during pregnancy that required hospitalization. All births were by cesarean section, with indications for planned deliveries being cephalopelvic disproportion and/or previous section. Important considerations for cesarean section in CHH females include reports of difficulties either with epidural anesthesia or with extraction of the baby during the delivery.

In 11 young adult males with CHH, blood and semen samples were evaluated (110). Serum levels of testosterone, inhibin B, and gonadotropins were normal, whereas semen analysis was aberrant in all and included low sperm concentration, decreased motility, and/or morphological abnormalities.

### Other clinical features

Despite the term “CHH,” 7–25% of patients have normal hair (11, 19, 65). In others, hair hypoplasia can be noted as thin and/or sparse hair, up to total alopecia (65).

Oral health has been assessed in a case-control study of 23 Finnish individuals (111). Patients presented more mucosal lesions and deep periodontic pockets. The prevalence of human papillomaviruses was similar in patients and controls, but bacterial microbiota diversity, richness, and microbial composition were different.

Structural abnormalities in thymus have been described in biopsies of two patients with CHH and Omenn syndrome, including loss of corticomedullary demarcation, signs of dysplasia, and depletion of Hassall corpuscles (19). In one of these patients, thymic epithelial cells were immature with lack of *Aire* expression, thymic dendritic cells were absent, and thymic Foxp3^+^ cells were markedly depleted (112). Another report on a fetus with CHH revealed a hypoplastic thymus with loss of cortex and medulla demarcation, absence of Hassall corpuscles, and a marked depletion of lymphocytes (103).

### AD

AD is a rare condition, with only few patients reported so far, and is characterized by extremely severe skeletal phenotype, the tallest patient having a height of 83 cm at the age of 19 years (66). In addition, patients with AD manifest mild mental retardation and hypodontia, which are not features of CHH (56). Radiographic signs in AD also differ from CHH: apart from metaphyseal changes, femoral epiphyses, pelvis, and vertebrae are also involved (113).

The immunologic phenotype in AD has been reported in a single case: an 11-year-old patient had normal results for immunoglobulin levels, lymphocyte subset counts, and blastic transformation (114). In addition, there is a mention of recurrent pneumonias in one of the patients, leading to lethal outcome, that were attributed to thoracic deformity (56).

The missense variants in *RMRP* reported in patients with AD phenotype only mildly affected cell growth and did not alter 5.8S rRNA levels when introduced into yeast (56). However, patient fibroblasts did exhibit poor growth and an increase in pre-5.8S rRNA, which were both reverted with transfection of fibroblasts with wild-type *RMRP*. In a comparison of human fibroblasts transfected with wild-type, the n.71A>G variant or the AD variant, cell proliferation, and rRNA cleavage rates were impaired in both variants, with more pronounced changes in case of the AD variant.

### Laboratory features of immunodeficiency

Lymphopenia is a common but not universal feature in CHH, present in 36–83% of patients from non-HSCT cohorts. All lymphocyte subsets, except for natural killer cells, are usually depleted but do not decline with age, as opposed to healthy children (84). Single patients also have neutropenia, either intrinsic or autoimmune (65, 67, 88, 115). A case of severe agranulocytosis requiring HSCT has been reported (11).

T cell counts are often low, and T cell repertoire is mostly restricted but may also be normal (19, 67, 116). The number of regulatory T cells is usually normal, and none of the 11 tested adults had double-negative T cells over 2% of total CD3^+^ cells (115). Naive T cell and recent thymic emigrants’ counts have only rarely been reported from CHH patients, ranging from being low in all (18/18, 100%) (58) to being normal in a significant proportion of patients (15/25, 60%) (84). Lower naive T cell and recent thymic emigrants’ counts have been associated with a more severe clinical immunodeficiency (84, 115). However, several children with CHH and profound, leaky SCID level T cell lymphopenia have been reported to remain well for decades with no interventions (84).

While B cell counts are often decreased, hypogammaglobulinemia is rare in patients with CHH; moreover, some individuals have hypergammaglobulinemia (84, 95, 115, 117). Naive and switched memory B cells are diminished in the majority of the patients tested (6/11, 55%, and 34/51, 67%, respectively), whereas activated B cells (CD21^low^CD38^low^) are increased (11/11, 100%) (115).

### Newborn SCID screening

T cell receptor excision circles (TREC) screening for SCID detects some newborns with CHH (116, 118). After implementation of SCID screening in Finland in 2019, several CHH newborns with reduced, but not absent, TREC have been identified (84), while others had normal TREC levels. Importantly, TREC levels can decrease from normal to undetectable in some CHH patients (19). In a study of eight CHH patients from the United States with abnormal SCID screening, all homozygous for the n.71A>G variant, four had decreased and four had absent TREC (119). Anemia requiring transfusion and HD were only noted in patients with absent TREC. However, infection susceptibility or birth length did not differ between these groups. All patients were alive and well with a median follow-up of 19 mo, despite that only two of them (with absent TREC) underwent HSCT. In a longitudinal Finnish study reporting seven children with CHH and TREC values measured at 1 day to 14 years of age, four had low and three had absent TREC (84). Absent, but not low TREC levels associated with the development of opportunistic infections during follow-up.

The absence of severe infections in several reported CHH newborns with absent TREC can be explained by multiple reasons. First, early HSCT in few reported and probably many unreported CHH infants prevents infections. In addition, the untransplanted children may still develop severe and/or opportunistic infections later, as described in an abovementioned Finnish study with longer follow-up. Finally, some children might have sufficient numbers of naïve T cells with normal function despite initial abnormal TREC counts. Therefore, individualized approach in management of CHH newborns with absent TREC is warranted.

### Vaccine safety and effectiveness

Serologic responses to protein vaccines are mostly normal in CHH, despite significant T and/or B cell lymphopenia (84). Responses to mumps, measles, rubella, and varicella after disease or after immunization are strong and long-lasting (97). Of the eight subjects evaluated for polysaccharide vaccine responses, seven had been diagnosed with specific antibody deficiency, and this has been suggested as a marker of a more severe clinical immunodeficiency (115).

While CHH is considered a combined immunodeficiency with contraindication for receiving live vaccines, in some mildly affected patients immunization may be safe and effective. In a retrospective health records review of Finnish CHH patients, 86/90 (96%) have received immunization with at least one live vaccine, with no documented complications or hospitalizations, not even following smallpox, polio, or yellow fever vaccine administration (97). However, a case of lethal vaccine-related paralytic poliomyelitis in an infant with clinical CHH diagnosis has been reported (120). Additionally, skin granulomas caused by vaccine-derived rubella virus have since been reported in a Finnish CHH child with severe immunodeficiency, who received rubella vaccination prior to immunological assessment, as well as in one non-Finnish adult with CHH (84, 106).

Five patients with CHH have been reportedly vaccinated with live varicella vaccine with no adverse events, and another five have been administered live varicella vaccine as a part of clinical trial (97). All trial participants had no clinical symptoms of immunodeficiency and had normal lymphocyte proliferative responses despite lymphopenia in three of them. The four immunized children had no adverse events, while adult patient developed a rash and knee swelling 3 wk after immunization that disappeared spontaneously. Cellular response to varicella vaccine was measured by interferon gamma ELISpot antigen assay from PBMC, and all five patients demonstrated induction of cellular immunity similar to the healthy control, four after the first dose.

### Clinical course and mortality

Multiple patients with CHH who live into late adulthood have been described, and in terms of immunodeficiency, they stay clinically asymptomatic despite significant lymphopenia (55, 95). However, there is an accumulation of clinical features over time, underscoring the importance of regular follow-up of patients with CHH even when asymptomatic (95).

The causes of death in patients with CHH reported in 1993 and related to CHH were mostly infections (*N* = 6, including pneumonia, colitis, septicemia, encephalitis, and tuberculosis), but also anemia (*N* = 3), lymphomas (*N* = 2), and HD (*N* = 2) (65). The mortality in CHH patients is increased compared with the general population, as demonstrated in 2001 (*N* = 120, seven CHH-related deaths) and 2019 (*N* = 80, 15 CHH-related deaths) for the Finnish cohort (95, 121). Overall SMR was 9.3 and 7.0 in 2001 and 2019, respectively, with infections being the main cause of deaths in children, whereas adults succumbed to malignancies, pulmonary diseases, and pneumonia. The median age at death was 41 years, being 24 years for infections, 41 years for malignancies, and 53 years for lung disease. The SMR of siblings and parents did not differ from the general population.

The longitudinal follow-up report of 80 Finnish patients with CHH demonstrated significant risk factors for early mortality (95). These included HD, pneumonia in the first year, as well as recurrent pneumonia or autoimmunity in adulthood. In addition, severe short stature below −4.0 SDS at birth significantly increased the probability of death, with SMR of 22 and SMR/SMR ratio of 5.4 for normal versus severely short birth length. Consistently, in the Amish CHH cohort, significantly shorter birth length was observed in patients with the most severe immunodeficiency phenotype (88). The more severe growth failure is associated with weaker lymphocyte proliferative responses to mitogens (122), as well as with the lower total lymphocyte and T cell counts (84). Recently, machine learning approach identified shorter birth length as a risk factor for the development of infections in CHH (84).

An international registry study described 74 subjects with CHH from the European Society for Immunodeficiencies Registry (*N* = 11), the United States Immunodeficiency Network Registry (*N* = 16), and the Finnish Skeletal Dysplasia Register (*N* = 47, different form the abovementioned 80 patients in the longitudinal Finnish study) (123). Six patients (6/74, 8%) experienced fatal outcome; three of them were from outside Finland. Causes of death included infections in adolescence, lymphomas in young adults, but also pulmonary complications after HSCT. Shorter birth length could not be confirmed as a risk factor for mortality in this study due to data unavailability. In conjunction with previous observations (95), opportunistic infections, specifically uncontrolled EBV infection, associated with increased mortality.

### HSCT

In CHH, HSCT has been performed for clinically obvious combined immunodeficiency, preemptively after abnormal TREC screening, for severe anemia without immunodeficiency, as well as for agranulocytosis (11, 67, 84, 119). Currently, there are geographical differences in HSCT strategies in CHH, which underscores the variability of the clinical manifestations and uncertainty in management. HSCT seems to be undertaken in the United States more often and at an earlier age, possibly as a consequence of newborn screening for SCID (123).

Immune reconstitution has been successful after HSCT in CHH, as well as the correction of anemia. Skeletal features are not altered by HSCT (68); however, two patients normalized their growth after HSCT, one homozygous and another compound heterozygous for promoter region duplications (19).

Several case series of patients undergoing HSCT for CHH have been published, underscoring the high prevalence of severe infectious complications after HSCT (Table S2). In a European series of 16 patients treated with HSCT between 1991 and 2006, at the age of 8 mo to 16.8 years, the majority received myeloablative conditioning (68). Overall survival (OS) was 63% (10/16) at a median follow-up of 7 years, with all survivors having normal immunity and being well, with no cases of grade 3–4 graft-versus-host-disease (GVHD). Six patients died, five of them due to infections. Full donor chimerism was achieved in 8/12 (67%) of patients. Importantly, severe infectious complications after HSCT occurred in patients despite full donor chimerism.

Another series described 13 patients from the United Kingdom, aged 1.5–125 mo (67). Only three received myeloablation, and OS was 85% (11/13) at a median follow-up of 50 mo. Also in this cohort, viral infections were the most common transplant-related complication. There were two deaths, one from infection. Full donor chimerism was reported in 73% (8/11). All 11 subjects had normal T cell reconstitution, and 82% (9/11) were off IGRT. A single subject had developed grade two chronic skin GVHD.

Finally, a series from the United States described six patients who underwent HSCT after reduced intensity conditioning at a median age of 10 mo (69). Five of the patients were alive and well at the median of 4.5 years follow-up (OS of 83%), with mixed chimerism and good T cell reconstitution, and four did not need IGRT anymore. The only patient in this series with HD died at day 85 after transplant from a multiorgan failure secondary to sepsis, acute respiratory distress syndrome, ileus, abdominal compartment syndrome, and thrombotic microangiopathy. Another patient developed chronic skin GVHD. The high rates of mixed chimerism did not preclude B cell reconstitution. Further studies are needed to determine the consequences of mixed chimerism on the risk of malignancies and other complications.

## Conclusions and future directions

Much has been learned about *RMRP* deficiency during the 60 years since the first description of CHH. These advancements help patients and clinicians in making management decisions and arranging multidisciplinary care and follow-up. However, many aspects of CHH require further study.

Most importantly, specific diagnostic tests for *RMRP* deficiency are still lacking, complicating the evaluation of *RMRP* variants. One could probably use any type of patient cell culture to explore the ubiquitous cell proliferation defect, but such test would neither be sensitive nor specific. Although normally proliferating cells would significantly reduce the possibility of *RMRP* deficiency, some patients with CHH have normal lymphocyte proliferative responses. On the other hand, the cell proliferation defect in *RMRP* deficiency makes other functional diagnostics challenging, as one has to differentiate whether the mechanism of any observed abnormality stems in the intrinsic *RMRP* deficiency or is secondary to the nonspecific impaired cell functioning.

We are in need of understanding the phenotypic variability in CHH to develop tools for predicting the individual clinical course and providing personalized management. As many patients with CHH are alive and well in their late adulthood, the decision on management of a clinically asymptomatic CHH newborn with lymphopenia is not trivial. The dose dependency in *RMRP* deficiency is an intriguing hypothesis and deserves further exploration. With the much-awaited recent discovery of *RMRP*-specific proteins, these studies are hopefully approaching. Also, the development of a diagnostic scoring system comprising genetic, clinical, biomarker, and laboratory features may further assist management.

In addition, understanding of the yet unidentified mechanisms of comorbidities in CHH, such as HD, anemia, and lymphomas, is expected to improve the management of these conditions, contributing to improved survival of CHH patients.

Finally, while HSCT is curative for severe anemia and immunodeficiency in CHH, the complications of HSCT are devastating for some patients. The selection of patients and optimal moment for HSCT, as well as considerations for improved infectious prophylaxis, should become a priority. Longitudinal prospective studies should be undertaken to compare HSCT strategies and indications; however, with the rarity of CHH, these would necessitate an international collaboration effort. Given the high prevalence of the founder variant and the sufficiency of the correction of a single nucleotide on one *RMRP* allele, gene therapy is an appealing management strategy in CHH awaiting research effort.

## Online supplemental material

Table S1 shows the summary of the studies describing the effects of *RMRP* deficiency. Table S2 shows the clinical and laboratory features, as well as outcomes, in patients with CHH reported in case series.

## Supplementary Material

Table S1 shows the summary of the studies describing the effects of *RMRP* deficiency.

Table S2 shows the clinical and laboratory features, as well as outcomes, in patients with CHH reported in case series.
