# Supplementary material for: Cartilage-hair hypoplasia: A comprehensive review
Source: J Hum Immun. 2025 Oct 1;1(4):e20250142. doi: 10.70962/jhi.20250142 (PMC13177382; doi:10.70962/jhi.20250142)
Supplement: Table S1 — shows the summary of the studies describing the effects of RMRP deficiency. [file jhi_20250142_tables1.docx]

Table S1. Summary of the studies describing the effects of *RMRP* deficiency.

|  | ***RMRP* expression** | **rRNA processing** | **Cell proliferation** | **Apoptosis** | **Chondrocyte function** |
| --- | --- | --- | --- | --- | --- |
| **Cell lines** |  |  |  |  |  |
| HeLa (n.71A>G) (38) | ↓ 30-50% | NA | NA | NA | NA |
| HeLa (indels/deletion of *RMRP*) (51) | NA | Accumulation of 5.8S and ITS1 | ↓ | NA | NA |
| NIH3T3 (n.71A>G) (38) | ↓ 30-50% | NA | NA | NA | NA |
| Mouse ES (n.71A>G) (38) | ↓ 20-30% | NA | NA | NA | NA |
| Mouse T cells (31) | NA | Delayed cleavage at ITS1 | ↓ | NA | NA |
| HEK293T (various variants including n.71A>G) (28) | ↓ | NA | NA | NA | NA |
| K562 (n.71A>G) (31) | Normal | Reduced amount of rRNA | NA | NA | NA |
| ATDC5 (*Rmrp* knockdown) (40) | NA | NA | ↓ | NA | Disrupted differentiation and mineralization; reduced expression of *Runx2, Col10a1, Alpl, Sox9, Col2a1;* increased expression of *Bapx1,* *Pthrp* |
| Human fibroblasts (various variants including n.71A>G) (56) | NA | NA | ↓, decreased *CCNA2* and increased *CCNB2* expression | NA | NA |
| **Yeast** |  |  |  |  |  |
| *nme1* promoter duplications (37) | ↓ | NA | NA | NA | NA |
| Depressed *nme1* transcription (33) | NA | Disrupted for 5.8S | NA | NA | NA |
| *nme1* mutagenesis | NA | Disrupted for 5.8S (50) | ↓ (34, 50, 57)  Delayed exit from mitosis, increased *Clb2* levels (57) | NA | NA |
| *nme1* (analogue for n.71A>G) (37) | NA | Disrupted | NA | NA | NA |
| **Animal models** |  |  |  |  |  |
| Zebra-fish (13-bp deletion of *rmrp*, including the site of n.71A>G) (39) | ↓↓↓ | NA | ↓, upregulation of *ccng1, ccnd1* and *cdkn2a/b* | ↑, upregulation of *tp53, mdm2* and *caspase8* | Disordered chondrocyte arrangement, delayed bone ossification, upregulation of Wnt/β-catenin pathway |
| **Patient-derived cells** |  |  |  |  |  |
| Fibroblasts | ↓ (40, 41) | Disrupted (31) | ↓ (55, 59)  Delayed progression from G2 to G1 (59) | NA | Impaired induction of *COL2A1* and *ALPA*, upregulation of PTHrP, disturbed hypertrophic differentiation (40).  Delayed engagement into transdifferentiation, disrupted terminal differentiation, altered expression of growth factors (41). |
| Lymphocytes | NA | NA | ↓ (42, 55) | ↑ , increased CD95 and CD95L, decreased Bcl-2, Bax and IAP expression (60) | NA |
| Peripheral blood mononuclear cells | ↓ (37, 42) | NA | Increased proportion of cells in G2/M phase (58) | ↑ (58) | NA |
| Whole blood (21) | ↓ | NA | NA | NA | NA |
| EBV-transformed lymphoblasts (38) | ↓↓ | NA | NA | NA | NA |

EBV Epstein-Barr virus, NA not assessed
