# Supplementary material for: Cartilage-hair hypoplasia: A comprehensive review
Source: J Hum Immun. 2025 Oct 1;1(4):e20250142. doi: 10.70962/jhi.20250142 (PMC13177382; doi:10.70962/jhi.20250142)
Supplement: Table S2 — shows the clinical and laboratory features, as well as outcomes, in patients with CHH reported in case series. [file jhi_20250142_tables2.docx]

Table S2. The clinical and laboratory features, as well as outcomes, in patients with cartilage-hair hypoplasia reported in case series. Importantly, there is an overlap between cohorts, and some of the patients are included in multiple publications.

| First author and publication year | Mäkitie, 1993 (65) | Mäkitie, 1998 (122) | Bonafe, 2005 (18) | Guggenheim, 2006 (124) | Hermanns, 2006 (76) | Kavadas, 2008 (19) | Rider, 2009 (88) | Bordon, 2010 (68) |
| --- | --- | --- | --- | --- | --- | --- | --- | --- |
| Number of patients | 108 | 35 | 36 | 3 | 22 | 12 | 25 | 16 |
| Geographical background | Finland | Finland | Europe, others | Canada | USA, Europe | Canada, Italy | Amish | Europe |
| *RMRP* n.71A>G/ n.71A>G | NR | NR | 5/36 (14%) | 0/3 (0%) | 4/22 (18%) | 1/12 (8%) | 25/25 (100%) | 2/12 (17%) |
| Age, years | 0.9-52 | 0.1-56 | 0.6-29 | 6-21 | 0.6-29 | 0.2-44 | 0.8-21 | 0.7-19 |
| Hair hypoplasia | 81/87 (93%) | NR | 11/36 (31%) | 3/3 (100%) | 19/19 (100%) | 9/12 (75%) | NR | NR |
| Short stature | 87/87 (100%) | NR | 36/36 (100%) | 3/3 (100%) | 22/22 (100%) | 11/12 (92%) | 25/25 (100%) | NR |
| Radiologic skeletal abnormalities | NR | NR | 34/36 (94%) | 3/3 (100%) | 20/20 (100%) | 4/7 (57%) | NR | NR |
| Hirschsprung’s disease | 8/108 (7%) | NR | 4/36 (11%) | NR | NR | NR | 3/25 (12%) | NR |
| No increased susceptibility to infections^1^ | 45/103 (44%) | 20/35 (57%) | 18/36 (50%) | 1/3 (33%) | 6/11 (55%) | 4/12 (33%) | 17/25 (68%) | NR |
| Severe or unusual infections | Severe varicella | None | NR | Pn (PJ) | Severe varicella | Pn (CMV, PJ, *Aspergillus*) | Diss HSV, Pn (CMV) *Haemophilus* meningitis | Severe varicella, persistent EBV viremia; adeno hepatitis |
| Recurrent pneumonia | NR | NR | NR | NR | NR | 1/12 (8%) | NR | 4/16 (25%) |
| Bronchiectasis | NR | NR | NR | NR | NR | 1/12 (8%) | NR | 6/16 (38%) |
| Rec OM and/or rec Sin | NR | NR | NR | NR | NR | 1/12 (8%) | 6/25 (24%) | 1/16 (6%) |
| Omenn syndrome | NR | NR | NR | 2/3 | NR | 4/12 (33%) | NR | 2/16 (13%) |
| Autoimmunity | NR | NR | 3/36 (8%) (AIHA) | NR | NR | NR | 3/25 (12%) (AIHA, ITP, JRA) | 3/16 (19%), (enteropathy, AIHA, hypothyroidism) |
| Lymphopenia | 51/79 (65%) | 12/33 (36%) | NR | NR | NR | 10/12 (83%) | In majority | 15/16 (94%) |
| Low T cells | NR | 15/29 (52%) | NR | 3/3 (100%) | 6/9 (67%) | 12/12 (100%) | NR | 15/16 (94%) |
| Low RTE or naive T cells | NR | NR | NR | NR | NR | NR | NR | NR |
| Reduced lymphocyte response to PHA | 53/60 (88%) | 22/32 (69%) | NR | 3/3 (100%) | 2/2 (100%) | 12/12 (100%) | In majority | 14/15 (93%) |
| Low TREC | NR | NR | NR | NR | NR | 5/5 (100%)^2^ | NR | NR |
| Skewed T cell repertoire | NR | NR | NR | NR | NR | 8/9 (89%) | NR | NR |
| Low B cells | NR | 2/23 (9%) | NR | 1/3 (33%) | NR | 6/12 (50%) | NR | 2/15 (13%) |
| Hypogammaglobulinemia | NR | 0/16 (0%) | NR | 1/3 (33%) | NR | 3/11 (27%) | In some | 4/15 (27%) |
| Low NK cells | NR | 0/15 (0%) | NR | 1/3 (33%) | NR | 2/12 (17%) | NR | 2/13 (15%) |
| Fatal outcome | 16/108 (15%) | NR | NR | 0/3 (0%) | NR | 1/12 (8%) | 1/25 (4%) | 5/15 (33%) |
| Causes of death | Multiple^3^ | NA | NA | NA | NA | Infections pre-HSCT | AIHA post-HSCT | All post-HSCT: infections, ITP |
| HSCT performed | NR | NR | NR | 3/3 (100%) | NR | 6/12 (50%) | 2/25 (8%) | 16/16 (100%) |
| HSCT complications | NA | NA | NA | 2/3 (67%) | NA | 1/6 (17%) | 2/2 (100%) | 12/16 (75%) |
| Description of HSCT complications | NA | NA | NA | Mild aGVHD | NA | Nephrotic syndrome | Fatal AIHA, chronic ITP | Multiple^4^ |

AIHA autoimmune hemolytic anemia; CMV cytomegalovirus; Diss disseminated; EBV Epstein-Barr virus; GVHD graft-versus-host-disease (a acute, c chronic); HSCT hematopoietic stem cell transplantation; HSV herpes simplex virus; ITP autoimmune thrombocytopenia; JRA juvenile rheumatoid arthritis; NA not applicable; NR not reported; OM otitis media; PJ *Pneumocystis jiroveci*; PHA phytohaemagglutinin; Pn pneumonia, RTE recent thymic emigrants; Sin rhinosinusitis; TREC T cell receptor excision circles; USA United States of America.

^1^ Variable definitions of increased susceptibility to infections have been used in different studies.

^2^ In a single patient TREC dropped from normal to undetectable on repeated measurements.

^3^ Infections, including pneumonia, colitis, septicemia, encephalitis and tuberculosis; anemia; lymphomas; Hirschsprung’s disease.

^4^ Within 1 year post-HSCT in 11 out of 16 patients: pulmonary hemorrhage and fatal multiple organ failure D32 (N=1), pneumonitis (N=1), cutaneous *Candida* infection (N=1), sepsis and lung aspergillosis and hemorrhagic BK-virus cystitis (N=1), mild cutaneous aGVHD (N=1), lung aspergillosis and candidiasis and gastrointestinal CMV and fatal sinocerebral mucormycosis (N=1), mild aGVHD and mild veno-occlusive disease (N=1), grade II aGVHD (N=1), engraftment failure and fatal adeno (N=1), engraftment failure and *Klebsiella* sepsis and fatal adeno (N=1), EBV post-transplant lymphoproliferative disorder (N=1). Beyond 1 year post-HSTC in 3 out of 12 patients: fatal ITP, acute nephritis, fatal pneumococcal sepsis.

Table 2 continued.

| First author and publication year | de la Fuente, 2011 (58) | Moshous, 2011 (89) | Faitelson, 2015 (90) | Ip, 2015 (67) | Aubert, 2017 (42) | Kostjukovits, 2017 (115) | Fitch, 2022 (69) | Pello, 2024 (84) |
| --- | --- | --- | --- | --- | --- | --- | --- | --- |
| Number of patients | 18 | 4 | 5 | 13 | 15 | 56 | 6 | 32 |
| Geographical background | Amish, others | France | Canada | United Kingdom | USA | Finnish | USA | Finnish |
| *RMRP* n.71A>G/ n.71A>G | 14/18 (78%) | 0/2 (0%) | 3/5 (60%) | 2/13 (15%) | 15/15 (100%) | 43/56 (77%) | 3/6 (50%) | 25/32 (78%) |
| Age, years | 1.0-21 | Children | Children | 0-9 yrs | 0.4-46 | 0.7-68 | 0.9-7 | 2.7-22 |
| Hair hypoplasia | NR | 4/4 (100%) | 2/2 (100%) | 6/13 (46%) | NR | NR | NR | NR |
| Short stature | NR | 4/4 (100%) | 2/2 (100%) | 13/13 (100%) | NR | NR | NR | NR |
| Radiologic skeletal abnormalities | NR | 4/4 (100%) | 3/3 (100%) | 7/9 (78%) | NR | NR | NR | NR |
| Hirschsprung’s disease | NR | NR | 0/5 (0%) | NR | NR | NR | 1/6 (17%) | 8/32 (25%) |
| No increased susceptibility to infections | 7/18 (78%) | 0/4 (0%) | 3/5 (60%) | 3/13 (23%) | 10/15 (67%) | 15/56 (27%) | 4/6 (77%) | 18/32 (56%) |
| Severe or unusual infections | EBV-LP, Pn (*Aspergillus*, CMV) | Severe HSV | Acute encephalitis | Diss adeno, CMV, EBV, HHV-6; EBV-LP; severe varicella | Pn (PJ), diss parvovirus | Severe varicella, severe warts | Pn (CMV) | Chronic norovirus GE, bocavirus hepatitis, refractory warts, RuV skin granulomas |
| Recurrent pneumonia | 1/18 (6%) | 1/4 (25%) | 1/5 (20%) | 1/13 (8%) | NR | 4/56 (7%) | 1/6 (17%) | 3/32 (9%) |
| Bronchiectasis | NR | 3/4 (75%) | 1/5 (20%) | 1/13 (8%) | 1/15 (7%) | 10/34 (29%) | NR | NR |
| Rec OM and/or rec Sin | NR | 1/4 (25%) | NR | 1/13 (8%) | 1/15 (7%) | NR | NR | 13/32 (41%) |
| Omenn syndrome | NR | NR | 1/5 (20%) | 1/13 (8%) | NR | NR | NR | NR |
| Autoimmunity | 1/18 (6%) (JRA) | 1/4 (25%) (AIHA) | 1/5 (20%) (AIHA) | 1/13 (8%) (Neutropenia) | 1/15 (7%) (JRA) | 1/56 (2%) (JRA) | NR | 1/32 (3%) (AIHA) |
| Lymphopenia | NR | 3/4 (75%) | NR | 13/13 (100%) | 10/15 (67%) | 31/56 (55%) | NR | NR |
| Low T cells | 10/18 (56%) | 4/4 (100%) | 5/5 (100%) | 12/13 (92%) | NR | 25/55 (45%) | NR | 14/32 (44%) |
| Low RTE or naive T cells | 18/18 (100%) | NR | NR | NR | NR | 27/52 (52%) | NR | 15/25 (60%) |
| Reduced lymphocyte response to PHA | 11/13 (85%) | 4/4 (100%) | 5/5 (100%) | 10/11 (91%) | NR | NR | NR | 8/32 (25%)^2^ |
| Low TREC | NR | NR | 2/4 (50%) | 9/9 (100%) | NR | NR | NR | 7/7 (100%) |
| Skewed T cell repertoire | NR | NR | 1/1 (100%) | 3/4 (75%) | NR | NR | NR | NR |
| Low B cells | 8/18 (44%) | 0/4 (0%) | 3/5 (60%) | 8/13 (62%) | NR | 37/55 (67%) | NR | 23/32 (72%) |
| Hypogammaglobulinemia | NR | 2/4 (50%) | 2/5 (40%) | 4/12 (33%) | 2/15 (13%) | 1/50 (2%) | NR | 1/32 |
| Low NK cells | NR | 0/4 (0%) | 0/5 (0%) | 1/12 (8%) | NR | 4/55 (7%) | NR | NR |
| Fatal outcome | NR | 1/4 (25%) | 2/5 (40%) | 2/13 (15%) | NR | 0/56 (0%) | 1/6 (17%) | 0/32 (0%) |
| Causes of death | NA | PML | Encephalitis, severe GVHD post-HSCT | All post-HSCT: infections, pulmonary hemorrhage | NA | NA | Post-HSCT sepsis | NA |
| HSCT performed | 1/18 (6%) | 2/4 (50%) | 3/5 (60%) | 13/13 (100%) | NR | 0/56 (0%) | 6/6 (100%) | 8/32 (25%) |
| HSCT complications | NR | NR | 2/3 (67%) | 7/13 (54%) | NA | NA | 5/6 (83%) | NR |
| Description of HSCT complications | NA | NA | Fatal GVHD, cGVHD with limb contractures | Multiple^3^ | NA | NA | Multiple^4^ | NA |

Adeno adenovirus; AIHA autoimmune hemolytic anemia; CMV cytomegalovirus; EBV Epstein-Barr virus; EBV-LP EBV-related lymphoproliferation; Diss disseminated; GE gastroenteritis; GVHD graft-versus-host-disease (a acute, c chronic); HHV-6 human herpes virus 6; HSCT hematopoietic stem cell transplantation; HSV herpes simplex virus; ITP autoimmune thrombocytopenia; JRA juvenile rheumatoid arthritis; NA not applicable; NR not reported; OM otitis media; PJ *Pneumocystis jiroveci*; PHA phytohaemagglutinin; PML progressive multifocal leukoencephalopathy; Pn pneumonia, RTE recent thymic emigrants; RuV vaccine-strain rubella virus induced; Sin rhinosinusitis; TREC T cell receptor excision circles; USA United States of America.

^1^ Variable definitions of increased susceptibility to infections have been used in different studies.

^2^ The measurement of lymphocyte proliferative responses to PHA was performed by the flow-cytometric assay for specific cell-mediated immune-response in activated whole blood.

^3^ CMV viremia and *Enterococcus* sepsis (N=1), EBV and adeno viremia and gastrointestinal grade III aGVHD (N=1), EBV viremia (N=1), grade II skin cGVHD skin (N=1), adeno viremia and astrovirus encephalopathy and fatal *Pseudomonas* pneumonia (N=1), pulmonary hemorrhage and fatal multiple organ failure (N=1).

^4^ Grade II skin aGVHD (N=2), grade II gastrointestinal aGVHD (N=1), AIHA (N=1), viremia (adeno, CMV, EBV) (N=5), sinusoidal obstruction syndrome (N=1), norovirus GE (N=1), fatal multiple organ failure and candidemia and ileus and abdominal compartment syndrome and acute respiratory distress syndrome and thrombotic microangiopathy (N=1).
